# Supplementary material for: The Hypothalamic–Pituitary–Thyroid Axis Equivalent in Normal and Cancerous Oral Tissues: A Scoping Review
Source: Int J Mol Sci. 2022 Nov 15;23(22):14096. doi: 10.3390/ijms232214096 (PMC9695915; doi:10.3390/ijms232214096)
Supplement: Supplementary file 1 [file ijms-23-14096-s001.zip › ijms-1987589-supplementary.pdf]

Supplementary Table S1. Cohen Kappa analysis

	B.Y Include	B.Y Exclude	S.X Include	S.X Exclude	J.Y Include	J.Y Exclude
L.W include	38	8	28	1	33	2
L.W exclude	0	15	1	30	2	10
Cohen's k	0.7002		0.933		0.776	

Supplementary Table S2. Difference in average TRH methylation levels between different groups in the THR gene sequence

Author (Year)	Cohort	Group (Sample): %TRH Methylation Average ± SD	*p*-Value
Puttipanyalears et al. (2018)	Not applicable: validation study	OSCC: 52.96 ± 5.36<br>Control: 5.7 ± 0.85	<0.001*
	Cohort 1	OSCC (oral rinse): 3.77 ± 0.60<br>Control (oral rinse): 2.66 ± 0.76	<0.001*
		OSCC (oral swab): 4.17 ± 0.58<br>OSCC (oral rinse): 3.77 ± 0.60	0.0012*
	Cohort 2	OSCC (oral rinse): 3.78 ± 0.48<br>Control (oral rinse): 2.86 ± 0.64	<0.001*
		Oropharyngeal SCC (oral rinse): 3.54 ± 0.37<br>Control (oral rinse): 2.86 ± 0.64	<0.001*
		OSCC (oral rinse): 3.78 ± 0.48<br>Oropharyngeal SCC (oral rinse): 3.54 ± 0.37	NS

* indicates statistically significant; NS indicates not statistically significant
